# Cognitive disability among older adults in Botswana: prevalence, trends, and sociodemographic determinants from cross-sectional data

**DOI:** 10.1186/s12877-025-06383-w

**Published:** 2025-08-29

**Authors:** Tiro Theodore Monamo, Mpho Keetile, Gobopamang Letamo

**Affiliations:** Department of Population Studies, University of Botswana, Private Bag, 00705 Gaborone, Botswana

**Keywords:** Cognitive disability, Older adults, Botswana, Prevalence, Socio-demographic determinants

## Abstract

**Background:**

Cognitive impairment is a critical issue among older adults, especially as populations age and demands on healthcare systems increase. In Botswana, the growing older adult population and the rising chronic diseases prevalence highlight the need for focused research on cognitive disability trends and their socio-demographic influences. By analysing demographic data and examining socio-demographic and health-related factors associated with cognitive disability, this research seeks to provide an in-depth understanding of the trajectory of cognitive disability within the ageing demographic in Botswana.

**Methods:**

This study analysed self-reported cognitive disability trends among older adults aged 65 + in Botswana using data from the 2017 Botswana Demographic Survey (BDS) and the 2022 Population and Housing Census (PHC). Disability prevalence was measured using the Washington Group Short Set (WGSS) questions. Bivariate analyses assessed prevalence and trends across demographic groups, and logistic regression models examined socio-demographic and health factors associated with cognitive disability.

**Results:**

The prevalence of self-reported cognitive disability in Botswana increased substantially over a five-year period, rising from 6.4% in 2017 to 22.2% in 2022—an increase of 15.8%. This upward trend was consistent across all sociodemographic indicators examined. The most rapid rise was observed among adults aged 80 years and older. In the 2022 data, several factors were significantly associated with higher odds of cognitive disability, including advancing age, female gender, residence in rural areas, unemployment, and the presence of physical health conditions (*p* < 0.05 for all associations).

**Conclusion:**

This study demonstrates a substantial and consistent increase in the prevalence of self-reported cognitive disability among older adults in Botswana between 2017 and 2022. This sharp rise likely reflects a combination of measurement differences, changing cultural attitudes, real epidemiological shifts associated with an ageing population and rising non-communicable diseases, and possibly the broader impacts of the COVID-19 pandemic. These findings underscore the urgent need for enhanced cognitive health interventions, improved data quality, and continued monitoring to address the growing burden of cognitive disability in Botswana’s ageing society.

**Supplementary Information:**

The online version contains supplementary material available at 10.1186/s12877-025-06383-w.

## Introduction

Cognitive impairment, encompassing memory loss, difficulties in decision-making, and challenges with basic cognitive functions, often impacts older adults’ quality of life, independence, and their need for long-term care [1]. In Botswana, this issue is particularly critical as the population of older adults grows [2], placing increased demand on healthcare and social support systems. The increase in the population of older adults, along with the rising prevalence of major chronic diseases and greater longevity among those suffering with many of these diseases, will lead to a growing number of functionally disabled older adults who may need long-term care (LTC) [3]. The rapid growth of the population aged 65 or older will lead to undesirable outcomes such as increased dependency, caregiver burden, and public healthcare costs [4]. Older adults in Botswana have traditionally relied on their families as the main source of LTC [5]. This situation is changing as fewer family members are available to provide care, due to shrinking family size caused by declining fertility rates combined with increased female workforce participation. Therefore, without a well-designed LTC system to address the growing care need, it is increasingly obvious that the current LTC situation in Botswana will not be sustainable in the coming years.

Identifying the levels and trends of cognitive disability is crucial for making informed assumptions about future cognitive disability patterns among the elderly [6]. While nationally representative longitudinal datasets are considered the gold standard for trend analysis, they are limited and not available in most low- and middle-income countries. However, similar studies are lacking in Botswana due to the absence of the necessary data. The existing literature offers limited insights into trends in the prevalence of cognitive disability, primarily because of the scarcity of research, the absence of nationally representative trend data, and the inconsistent definitions and measures of disability.

In response to this gap, this study aims to measure the levels, trends, and differentials of cognitive disability among older adults in Botswana. The analysis is based on cross-sectional data from the 2017 Botswana Demographic Survey and the 2022 Botswana Population and Housing Census. Given that the topic of cognitive disability among older adults in Botswana has not been systematically or comprehensively assessed, this study is essential for understanding the trends and dynamics of cognitive disability among the elderly.

Moreover, the sociodemographic determinants of cognitive disability among older adults have remained largely underexplored within the context of Botswana. This study seeks to bridge this critical gap by systematically examining the prevalence and temporal trends of cognitive disability using nationally representative datasets. By leveraging data from the 2017 Botswana Demographic Survey and the 2022 Population and Housing Census, the study not only quantifies changes in cognitive disability over time but also identifies key sociodemographic and health-related factors associated with its occurrence. In doing so, it provides a more nuanced understanding of the interplay between ageing, social disadvantage, health status, and cognitive outcomes, thereby offering evidence to inform targeted public health interventions and policy development aimed at improving the well-being of Botswana’s ageing population.

These insights are intended to support the development of policies and interventions that could improve cognitive health and quality of life for older adults, aligning with Botswana’s broader goals for sustainable and inclusive healthcare provision.

## Methods

### Study design

The analysis in this paper was based on data from the 2017 Botswana Demographic Survey (BDS) and the 2022 Botswana Population and Housing Census (PHC). Both the 2017 BDS and the 2022 PHC utilized the Washington Group Short Set (WGSS) of questions to measure disability. The WGSS, developed by the United Nations City Group, is designed to identify individuals who are at risk of participation restrictions due to health conditions or impairments. The tool focuses on six fundamental functional domains: seeing, hearing, walking, cognition, communication, and self-care.

The WGSS is widely recommended and utilized, including for disaggregating Sustainable Development Goal (SDG) indicators. It is valued for being simple, quick, reproducible, and easy to translate into various languages. The WGSS is particularly suitable for use in censuses and large-scale surveys where only a few questions can be included. Additionally, because the tool is widely used, its results can be compared across different time periods and between countries. Notably, the WGSS is non-stigmatizing as it does not directly inquire about disability. However, the WGSS has limitations. It provides limited data on other aspects of disability, such as participation restrictions or specific impairments, and it does not include information on psychosocial functioning or mental health [7].

Data from the 2001 and 2011 Population and Housing Censuses were not included in this analysis because, although these datasets are otherwise comparable to the 2022 census data, they did not incorporate the Washington Group Short Set (WGSS) of questions on disability. As a result, cognitive disability could not be measured consistently across all census years, and only the 2017 Botswana Demographic Survey and the 2022 Census data were utilized for this study.

### Data sources

The 2017 Botswana Demographic Survey (BDS) was an inter-censal survey conducted to update demographic indicators and provide reliable data for planning and policy development. Utilizing a stratified two-stage probability sampling design, the survey selected 478 enumeration areas (EAs) proportionally distributed across cities and towns, urban villages, and rural areas, based on the 2011 census sampling frame. Within each EA, a complete household listing was conducted, and 20 households were systematically sampled, resulting in a total sample of 9,560 households [8]. The 2017 BDS incorporated digital data collection methods to enhance data accuracy and management, although detailed response rates were not explicitly reported. In contrast, the 2022 Botswana Population and Housing Census (PHC) aimed for full population enumeration, ultimately recording 2,346,179 individuals. The 2022 PHC also employed advanced digital technologies throughout the data collection and processing phases, significantly improving data quality and expediting the release of results. Together, the rigorous, technologically supported methodology of the 2017 BDS and the comprehensive digital enumeration of the 2022 PHC provide a strong and comparable foundation for the analyses undertaken in this study.

### Study population and sample size

The data for this study was drawn specifically from individuals aged 65 years and older. For the 2017 Botswana Demographic Survey (BDS), the analysis was based on a subsample of 118,060 older adults extracted from the national dataset. It is important to clarify that the figure of 118,060 refers to the weighted national estimate of the older adult population, derived from the survey’s stratified two-stage probability sample, rather than the actual number of older adult respondents surveyed. The version of the dataset available for this study contained weighted population estimates only; the unweighted individual-level sample size was not included in the dataset provided by Statistics Botswana. Similarly, for the 2022 Botswana Population and Housing Census (PHC), 130,551 older adults were identified from the complete enumeration.

### Cognitive disability

Cognitive impairment and dementia are significant age-related public health concerns in the elderly population, often leading to disability, dependency, and a diminished quality of life [9]. Cognitive disability is generally defined as difficulty in remembering, learning new information, comprehending language, focusing attention, or making decisions that affect daily life [10].

In this study, cognitive disability was measured using a composite variable derived from two key questions, one assessing memory-related disability and the other communication-related disability. The first question, focused on memory, sought to identify individuals who have difficulty remembering or concentrating, which in turn impairs their ability to carry out daily activities. The second question addressed communication challenges, aiming to identify those who struggle with speaking, listening, or understanding speech, thereby affecting their ability to make themselves understood or to understand others.

Each question offered four response options: (1) no difficulty, (2) some difficulty, (3) a lot of difficulty, and (4) unable to perform the task at all. For the purpose of analysis, the responses were dichotomized. A response of “0” indicated no disability (no difficulty), while a response of “1” represented the presence of a disability, including those who reported some difficulty, significant difficulty, or total inability to remember or communicate effectively. This approach allowed for a clear identification of cognitive disabilities in the study population.

### Statistical analysis

This study examined the prevalence of cognitive disability and its association with sociodemographic factors among older adults aged 65 years and above, using data from the 2017 Botswana Demographic Survey and the 2022 Botswana Population and Housing Census.

The primary outcome variable was self-reported cognitive disability, and the exposures of interest were sociodemographic and health characteristics including age, sex, locality type, education level, employment status, marital status, religion and presence of other health conditions. Bivariate analyses were first conducted to estimate the prevalence of cognitive disability across these characteristics within each dataset. The Chi-squared test was used to assess differences in prevalence between groups, with a p-value of less than 0.05 considered statistically significant. Additionally, differences in cognitive disability prevalence between males and females were examined by age groups and by types of localities (cities and towns, urban villages, and rural areas).

For the multivariable analysis, separate multiple logistic regression models were fitted for each dataset year (2017 and 2022) to identify factors independently associated with cognitive disability. Adjusted odds ratios (AORs) and their corresponding 95% confidence intervals (CIs) were calculated to quantify the strength and direction of associations between sociodemographic characteristics and cognitive disability. All the variables were simultaneously included in each regression model to control for potential confounding effects. The odds ratios were obtained by exponentiating the logistic regression coefficients. A 5% significance level was applied to determine statistically significant associations.

Regarding trends, no formal statistical trend analysis (such as interaction terms or repeated measures models) was conducted. Instead, prevalence estimates were compared descriptively between 2017 and 2022 to illustrate apparent changes over the five-year period. All analyses were performed using IBM SPSS Statistics software, version 29.0.

## Results

### Completeness of the data on cognitive disability

The results in Table 1 below presents the completeness of data on cognitive disability for the 2017 Botswana Demographic Survey (BDS) and the 2022 Population and Housing Census (PHC). In the 2017 BDS, a total of 116,377 interviews were completed with no missing data, while 1,683 interviews had missing data, resulting in a high completeness rate of 98.6%. Similarly, the 2022 PHC included 125,810 complete interviews, with 4,741 interviews containing missing data, yielding a completeness rate of 96.4%.

The missing data in both the 2017 BDS and 2022 census were handled using listwise deletion, meaning that any records with missing data were excluded from the analysis. This approach was chosen due to the small proportion of missing data, which accounted for only 1.4% in the 2017 BDS and 3.6% in the 2022 PHC. Given the low level of missingness, listwise deletion was deemed appropriate, as it minimizes the risk of bias while ensuring that the remaining data used for analysis were complete and reliable. Moreover, the exclusion of such a small proportion of cases is unlikely to significantly affect the overall findings or introduce substantial bias into the results.

**Table 1:** Completeness of the data on cognitive disability

Dataset	Total Interviewed with no missing data	Missing data	Total interviewed with missing data	Completeness rate (%)
2017 BDS	116,377	1683	118,060	98.6
2022 PHC	125,810	4741	130,551	96.4

### Background characteristics of the study population

The study population consisted of 118,060 older adults in 2017 and 130,551 older adults in 2022. Results from Table 2 show that, for both 2017 and 2022, the highest proportion of older adults were in the 65–69 age group (32.0% in 2017 and 35.3% in 2022). This was followed by the 80 years and older group, accounting for 29.0% in 2017 and 25.7% in 2022. The 70–74 age group comprised 22.2% in 2017 and 23.6% in 2022, while the 75–79 age group represented the smallest proportion, with 17.2% in 2017 and 15.2% in 2022. In both 2017 and 2022, females made up 60% of the older adult population, while males accounted for 40%. Many older adults resided in rural areas during both years, with 51.2% living in rural areas in 2017 and 48.8% in 2022. A smaller proportion lived in urban areas (41.1% in 2017 and 42.7% in 2022), while the least resided in cities and towns (7.7% in 2017 and 8.5% in 2022). In terms of education, 72.5% of the older adults had primary education or less in both years. Additionally, around 77% of older adults were unemployed in both 2017 and 2022.

In the year 2017, various disabilities, indicate significant levels of limitations among older adults. Regarding vision, 40.7% of the population reported difficulty seeing, while 59.3% had no such issues. Hearing difficulties affected 16.3% of respondents, with the majority (83.7%) reporting no issues. Cognitive challenges were less prevalent, with 5.9% of older adults experiencing memory difficulties, while 94.1% had no such impairments. Communication difficulties were rare, affecting only 0.6% of the population, with 99.4% reporting no challenges in this area. Mobility impairment was reported by 26.4% of individuals, indicating a notable portion of the population facing difficulty walking, while 73.6% had no issues. Finally, difficulties in self-care were reported by 2.7% of the population, while 97.3% had no such limitations, suggesting that while most older adults could care for themselves, a small but significant fraction required assistance.

In 2022, 59.1% reported no difficulty seeing, while 40.9% experienced visual impairments. In terms of hearing, 78.8% of the population reported no difficulties, with 21.2% facing hearing challenges. Cognitive disabilities, particularly memory-related issues, affected 20.9% of respondents, while 79.1% had no such impairments. Communication difficulties were relatively uncommon, with only 4.0% reporting issues, and 96.0% of the population experiencing no communication challenges. Mobility impairment, measured by the ability to walk, was reported by 28.1% of the population, while 71.9% had no difficulty walking. Self-care limitations, a significant indicator of functional independence, affected 9.1% of older adults, while the majority (90.9%) were able to care for themselves without difficulty.

**Table 2:** Background characteristics of the study population

	2017		2022	
	Frequency (*N* = 118 060)	Percentage	Frequency (*N* = 130 551)	Percentage
**Socio-demographic**				
**Age-group**				
65–69	37,441	32.0	46,038	35.3
70–74	25,247	21.6	30,806	23.6
75–79	20,319	17.4	20,133	15.4
80+	34,054	29.0	33,574	25.7
Total	117,071	100	130,551	100
**Sex**				
Male	46,100	39.5	52,094	39.9
Female	71,427	60.5	78,457	60.1
Total	117,527	100	130,551	100
**Locality Type**				
Cities and towns	9036	7.7	11,100	8.5
Urban villages	48,556	41.1	55,802	42.7
Rural areas	60,468	51.2	63,649	48.8
Total	118,060	100	130,551	100
**Level of Education**				
Primary/less	48,339	72.5	50,295	74.9
Secondary	8568	12.9	9055	13.5
Tertiary/higher	9747	14.6	7770	11.6
Total	66,654	100	67,120	100
**Employment status**				
Employed	25,817	22.2	25,555	20.2
Not employed	90,275	77.8	100,760	79.8
Total	116,092	100	126,315	100
**Marital Status**				
Married	43,644	37.6	46,602	36.9
Never married	21,354	18.4	44,173	35.0
Living together	10,252	8.8	5657	4.5
Previously married	40,843	35.2	29,904	23.6
Total	116,093	100	126,336	100
**Religion**				
Christianity	98,698	85.1	107,685	84.9
African Tradition Religion	4374	3.8	8552	6.7
No religion	12,421	10.7	8912	7.0
Others	453	0.4	1739	1.4
Total	115,946	100	126,888	100
**Disabilities**				
**Difficulty in seeing**				
No	68,295	59.3	74,353	59.1
Yes	46,967	40.7	51,460	40.9
Total	115,262	100	125,813	100
**Difficulty in hearing**				
No	96,849	83.7	99,083	78.8
Yes	18,913	16.3	26,730	21.2
Total	115,762	100	125,813	100
**Difficulty in remembering**				
No	109,220	94.1	99,473	79.1
Yes	6893	5.9	26,337	20.9
Total	116,113	100	125,810	100
**Difficulty in communicating**				
No	115,670	99.4	120,734	96.0
Yes	707	0.6	5080	4.0
Total	116,377	100	125,814	100
**Difficulty in walking**				
No	85,100	73.6	90,442	71.9
Yes	30,524	26.4	35,370	28.1
Total	115,624	100	125,812	100
**Difficulty in self-care**				
No	112,954	97.3	114,319	90.9
Yes	3162	2.7	11,491	9.1
Total	116,116	100	125,810	100

All figures in the BDS 2017 represent weighted estimates of the national population. The dataset does not contain unweighted sample counts

### Prevalence and trends of cognitive disability by background characteristics

The prevalence of self-reported cognitive disability increased from the year 2017 to the year 2022. The overall rate was 6.4% in 2017 and increased to 22.2% in 2022. The change after 5 years was 15.8%. By age groups, the upward trend between the two periods existed across all the age groups. The prevalence of cognitive disability among older adults aged 80 years and above increased more rapidly compared to the other age groups. The change in prevalence of cognitive disability after 5 years in older adults aged above 80 years was 25.2%. By gender, the upward trend over time occurred in both males and females. The prevalence of cognitive disability in males increased more slowly than in females. The change in prevalence of cognitive disability after 5 years in males and females was 13.7% and 17.0%, respectively. All other groups under locality types, level of education, employment status, marital status and religion also experienced an increase in the prevalence of cognitive disability from the year 2017 to the year 2022.

Regarding disabilities, the prevalence of cognitive disability exhibited an upward trend across all factors between 2017 and 2022. An increase in cognitive disability prevalence was observed among older adults with and without disabilities in seeing, walking, hearing, and self-care; however, the rise was notably more pronounced among those with these disabilities. Among the health-related difficulties, older adults experiencing self-care limitations showed the highest five-year increase in cognitive disability prevalence (44.5%), followed by those with hearing difficulties (40.7%) and walking difficulties (40.0%). Detailed prevalence rates and trends are presented in Table 3.

**Table 3:** Prevalence of cognitive disability and its trends among older adults in Botswana

	2017 (%)	2022 (%)	5-year change (%)
**Overall Prevalence**	6.4	22.2	15.8
**Socio-demographic**			
**Age-group**			
65–69	5.3	13.7	8.4
70–74	4.8	18.7	13.9
75–79	3.8	23.4	19.6
80+	10.6	35.8	25.2
p-value	< 0.001	< 0.001	
**Sex**			
Male	3.9	17.6	13.7
Female	8.0	25.0	17.0
p-value	< 0.001	< 0.001	
**Locality Type**			
Cities and towns	3.9	15.1	11.2
Urban villages	6.2	21.1	14.9
Rural areas	6.9	23.9	17.0
p-value	< 0.001	< 0.001	
**Level of Education**			
Primary/less	5.9	21.8	15.9
Secondary	2.3	13.2	10.9
Tertiary/higher	2.1	10.0	7.9
p-value	< 0.001	< 0.001	
**Employment status**			
Employed	4.6	14.4	9.8
Not employed	8.7	24.0	15.3
p-value	< 0.001	< 0.001	
**Marital Status**			
Married	4.1	18.6	14.5
Never married	10.0	23.0	13.0
Living together	3.4	19.1	15.7
Previously married	7.7	26.8	19.1
p-value	< 0.001	< 0.001	
**Religion**			
Christianity	6.3	21.8	15.5
African Tradition Religion	6.2	24.5	18.3
No religion	8.0	23.2	15.2
Others	0.0	15.7	15.7
p-value	< 0.001	< 0.001	
**Disabilities**			
**Difficulty in seeing**			
No	4.6	10.7	6.1
Yes	8.7	38.3	29.6
p-value	< 0.001	< 0.001	
**Difficulty in hearing**			
No	5.6	14.3	8.7
Yes	9.8	50.5	40.7
p-value	< 0.001	< 0.001	
**Difficulty in walking**			
No	5.6	11.5	5.9
Yes	8.8	48.8	40.0
p-value	< 0.001	< 0.001	
**Difficulty in self-care**			
No	5.9	17.4	11.5
Yes	23.6	68.1	44.5
p-value	< 0.001	< 0.001	

### Variations of cognitive disability by sex and age groups

**Fig. 1:**
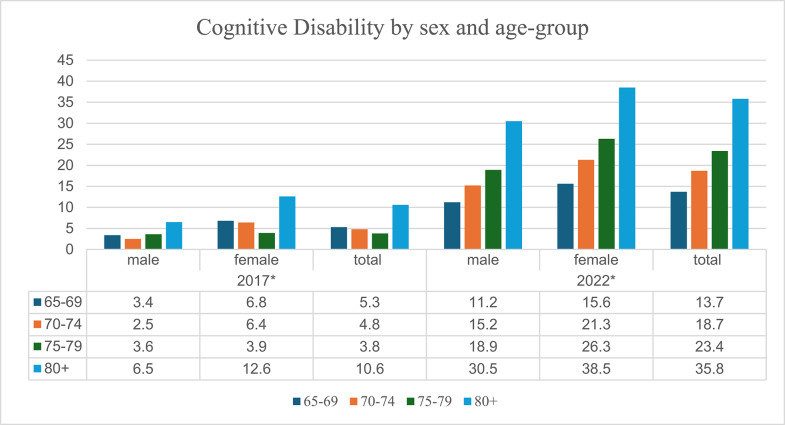
Cognitive Disability by sex and age-groups. ***p < 0.001, **p < 0.01, *p < 0.05

Figure 1 below demonstrates the differences of cognitive disability by sex and age groups. The statistics shows that cognitive disability rates are higher among females than males across all the age groups in both the year 2017 and 2022. In 2017, the rates of cognitive disability are higher among those aged 80 years and above, followed by those aged between 65 and 69 and those aged between 70 and 74 years. In 2022, the rate of cognitive disability increases with age. For those aged 80 years and above, the cognitive disability increased rapidly compared to the other age groups. Overall, there was an increase in cognitive disability rates across all the age-groups and sexes from the year 2017 to 2022.

### Variations of cognitive disability by sex and types of localities

**Fig. 2:**
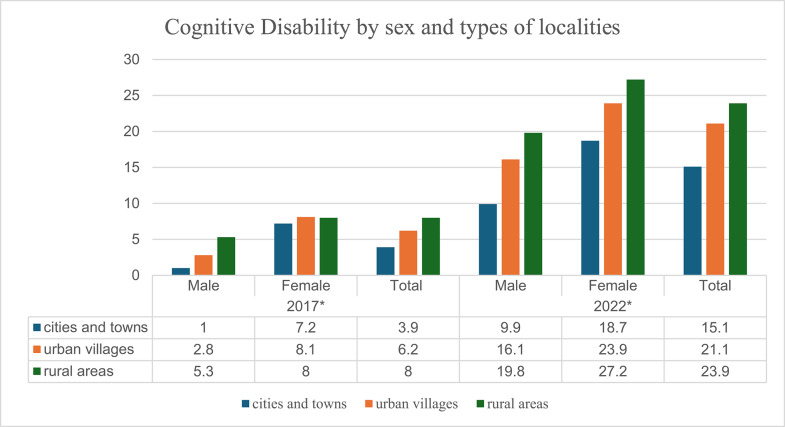
Cognitive Disability by sex and types of localities. ***p < 0.001, **p < 0.01, *p < 0.05

Figure 2 below displays the diagram of cognitive disability prevalence in 2017 and 2022 by sex and types of localities. The diagram depicts that the cognitive disability rates are higher among females than males in all the types of localities for both the years 2017 and 2022. For both the two periods, disability rates were higher among older adults who reside in rural areas for both sexes, followed by those who reside in urban villages and lastly those who live in cities and towns.

### Multivariate logistic regression analysis of cognitive disability

The results in Table 4 showed the differential influence of the socio-demographic and existing disabilities on cognitive disability between the years 2017 and 2022. In 2017, cognitive disability was significantly associated with all the socio-demographic and existing disabilities at p-value < 0.05, except for those aged 80 years and above and the living together socio-demographic groups. Socio-demographic factors strongly associated with cognitive disability in 2022 (all p-values < 0.05) were age 70–74 (OR = 1.124, p-value 0.001), age 75–79 (OR = 1.083, p-value = 0.05), age 80 and above (OR = 1.115, p-value = 0.001), female (OR = 1.288, p-value), urban villages (OR = 1.189, p-value = 0.001), rural areas (OR = 1.366, p-value = 0.001), not employed (OR = 1.125, *p* = 0.001), previously married (OR = 1.067, p-value = 0.05), and other religious affiliations (OR = 0.660, p-value = 0.001). In both 2017 and 2022, having an existing disability was significantly associated with higher odds of reporting cognitive disability, with p-values < 0.001 in each year.

**Table 4:** Logistic regression analysis of cognitive disability among older adults in Botswana

	2017 AOR (95% CI)	2022 AOR (95% CI)
**Socio-demographic**		
**Age-group**		
65–69	1	1
70–74	0.814*** (0.731;0.906)	1.124*** (1.060;1.193)
75–79	0.604*** (0.531;0.686)	1.083* (1.012;1.189)
80+	1.077 (0.968;1.198)	1.115*** (1.045;1.189)
**Sex**		
Male	1	1
Female	1.795*** (1.601;2.013)	1.288*** (1.221;1.359)
**Locality Type**		
Cities and towns	1	1
Urban villages	1.940*** (1.630;2.331)	1.189*** (1.097;1.289)
Rural areas	1.945*** (1.624;2.330)	1.366*** (1.257; 1.482)
**Level of Education**		
Primary/less	1	1
Secondary	0.731*** (0.660;0.825)	0.852*** (0.792;0.917)
Tertiary/higher	0.621*** (0.587;0.791)	0.718*** (0.657;0.784)
**Employment status**		
Employed	1	1
Not employed	1.214*** (1.089;1.354)	1.125*** (1.060;1.195)
**Marital status**		
Married	1	1
Never married	1.669*** (1.501;1.856)	0.962 (0.908;1.019)
Living together	1.231 (1.113;1.492)	0.969 (0.842;0.1.115)
Previously married	0.936* (0.820;1.001)	1.067* (1.007;1.130)
**Religion**		
Christianity	1	1
African Tradition Religion	2.652*** (2.036;3.453)	1.079 (0.958;1.216)
No religion	0.645*** (0.542;0.766)	0.899 (0.792;1.006)
Others	0.0	0.660*** (0.523;0.833)
**Disabilities**		
**Difficulty in seeing**		
No	1	1
Yes	1.558*** (1.437;1.690)	2.451*** (2.338;2.570)
**Difficulty in hearing**		
No	1	1
Yes	1.609*** (1.464;1.769)	2.700*** (2.564;2.844)
**Difficulty in walking**		
No	1	1
Yes	1.245*** (1.144;1.354)	3.282*** (3.127;3.445)
**Difficulty in self-care**		
No	1	1
Yes	6.852*** (5.748;8.168)	3.475*** (3.216;3.755)

***p < 0.001, **p < 0.01, *p < 0.05

## Discussion

The main finding of this study is that the prevalence of self-reported cognitive disability among older adults in Botswana was substantially higher in 2022 compared to 2017, with increases consistently observed across all sociodemographic categories. Specifically, the prevalence nearly quadrupled over the five-year period, a striking trend that demands careful interpretation. Several possible explanations can account for this remarkable rise. First, measurement differences and biases were considered. Although both the 2017 Botswana Demographic Survey (BDS) and the 2022 Population and Housing Census (PHC) employed the Washington Group Short Set (WGSS) of questions to assess disability, there were contextual and operational differences. The 2017 BDS was a nationally representative survey, whereas the 2022 PHC involved a full enumeration, both using digital data collection tools. While the WGSS questions remained conceptually similar, variations in interviewer training, administration procedures, or the reliance on proxy responses, especially for individuals with severe impairments, may have introduced response biases. Proxy reporting, more common in census data, can lead to both under- and overestimation depending on the proxy’s familiarity with the respondent’s cognitive condition [11].

Additionally, changing cultural perceptions and increased public awareness around disability may have encouraged greater self-identification. Botswana has taken significant steps towards promoting disability rights, including adopting the National Policy on Care for People with Disabilities and aligning with international frameworks such as the UN Convention on the Rights of Persons with Disabilities (CRPD). The Country acceded to the Convention on the Rights of Persons with Disabilities (CRPD) in 2021, thereby indicating its willingness to adhere to its norms and standards [12]. Around the same time, the Southern Africa Litigation Centre (SALC) applauded the Government of Botswana for ratifying CRPD after years of delay, despite intense advocacy by Civil Society Organizations working on disability rights in Botswana and the SADC region [13]. These shifts likely reduced stigma and encouraged older adults and their families to report cognitive difficulties more openly in 2022 compared to 2017. Furthermore, genuine epidemiological increases in cognitive impairment cannot be ruled out. Botswana’s ageing population is growing, and the prevalence of non-communicable diseases (NCDs) such as hypertension, diabetes, and stroke continues to rise [14], all of which are well-known risk factors for cognitive decline [15]. Coupled with longer life expectancy and changing health profiles, the country may indeed be witnessing a real increase in cognitive disability burden among older adults.

Importantly, the aftermath of the COVID-19 pandemic may have had a contributing factor. Emerging evidence globally suggests that COVID-19 and its long-term health impacts, including “long COVID” neurological symptoms, have disproportionately affected older populations [16]. While data from high-income countries show increased mental health challenges post-COVID, there is limited empirical research from Sub-Saharan Africa. Therefore, the potential contribution of COVID-19 in this context remains speculative. In Botswana, older adults may have experienced increased cognitive decline due to direct viral effects, disruptions in healthcare access, increased isolation, and mental health challenges during the pandemic period. The social and economic stressors of the pandemic may have exacerbated existing vulnerabilities, contributing to the observed rise in cognitive disability between 2017 and 2022.

An unexpected observation in this study was that disability prevalence did not consistently increase with age in the 2017 dataset, which is highly unusual and deviates from well-established patterns in the global literature. Typically, disability prevalence is expected to rise with advancing age, as older individuals face cumulative exposure to health risks, biological degeneration, and chronic conditions [17]. Several potential explanations could account for this atypical pattern in the 2017 Botswana Demographic Survey data. First, issues related to data quality were considered. Underreporting or misclassification of cognitive disability among the oldest age groups could have occurred due to difficulties in self-reporting, cognitive limitations of respondents, or the absence of proxy respondents for those unable to answer for themselves. If older adults with more severe impairments were less likely to participate in the survey or if their disabilities were under-identified by interviewers, the prevalence rates would be artificially suppressed. Additionally, social and household dynamics could affect reporting. Older adults living in multigenerational households may have received greater informal support that masked or compensated for functional limitations, leading respondents or proxy reporters to underestimate the presence of cognitive difficulties.

The findings of this study also showed that being 80 years and above was significantly associated with the highest prevalence of cognitive disability among older adults in Botswana. There is a positive relationship between age and disability. The more an older adult gets, the more they are likely to report to being disabled [18]. This relationship can be explained by the long-term consequences of exposure to environmental risks and the effects of poor health behaviours that may have been experienced by older adults [19]. Older age is also characterized by the emergence of several complex health states such as hypertension, diabetes, heart disease, mobility issues, falls, vision loss, hearing loss, respiratory infections, urinary incontinence and dementia [20]. These underlying factors often lead to cognitive disability.

Another key finding of this study is the consistently higher prevalence of cognitive disability among older women compared to men in both 2017 and 2022, a pattern that aligns with a substantial body of international research documenting higher rates of disability among women across nearly all age groups. Several factors contribute to this gender disparity beyond demographic composition. Biologically, women generally live longer than men, increasing their exposure to the risk of age-related conditions such as cognitive decline [21]; however, the present finding relates to age-specific prevalence, meaning women are more likely to experience disability at any given older age. Additionally, women are more likely to suffer from multiple non-communicable diseases (NCDs) and multi morbidity [15], conditions that significantly elevate the risk of cognitive impairment.

In Botswana, older women have been shown to bear a disproportionate burden of chronic diseases compared to men [14], potentially exacerbating vulnerability to cognitive decline. Socioeconomic factors also play a crucial role: older women have historically had lower access to education, formal employment, and healthcare services, factors that accumulate over the life course and negatively impact cognitive resilience [22]. Psychosocial dimensions, such as higher rates of widowhood, social isolation, financial insecurity, and caregiving responsibilities, further contribute to the elevated prevalence of cognitive disability among women [23]. In the Botswana context, traditional gender roles often place emotional and caregiving labour disproportionately on women, increasing their stress and cognitive burden in later life. Furthermore, healthcare-seeking behaviour may also influence the observed differences; women are more likely than men to report health problems and access healthcare services [24], which could result in higher self-reporting of cognitive difficulties and partially inflate the gender disparity. Overall, the higher prevalence of cognitive disability among older women reflects a complex interplay of biological, social, economic, and behavioural factors rather than mere differences in population structure.

This study further revealed that educational attainment was strongly associated with cognitive disability in both 2017 and 2022. In both years, older adults with lower levels of education exhibited significantly higher odds of reporting cognitive disability. Specifically, individuals with only primary education or less faced the greatest risk, followed by those with secondary education, while those with tertiary or higher education consistently demonstrated the lowest odds of cognitive disability. Several explanations may account for this relationship. Higher educational attainment may enhance individuals’ ability to efficiently manage resources, navigate health systems, and apply health knowledge, ultimately supporting better cognitive outcomes. Cutler and Lleras-Muney [25] found that among elderly individuals with disabilities, those with higher education levels were less adversely affected by their impairments, often utilizing more assistive technologies and paid care services. Moreover, education plays a crucial role in facilitating economic participation and fostering social engagement, both of which are vital for coping with disability in later life [26]. Greater familiarity with institutional systems and heightened motivation, both linked to higher education, likely promote more active participation in social and economic spheres. Consistent with these findings, Brigola et al. [27] reported that lower levels of education were inversely associated with cognitive performance, functional independence, and resistance to frailty, underscoring the importance of investing in education throughout the life course as a preventative strategy against cognitive decline and disability in older age.

Currently, there is a notable lack of population-based data on the marital status and relationship patterns of older adults living with disabilities. This section explores marriage formation and dissolution among disabled older adults in Botswana. In 2017, the findings indicated that older adults who had never been married were more likely to report cognitive disability. However, by 2022, the pattern shifted, with previously married older adults (those separated, divorced, or widowed) exhibiting higher rates of cognitive disability. It is important to note that census data in Botswana do not capture detailed information on the number of marriages or common-law unions, limiting the ability of this study to determine whether disabled older adults are at greater risk of separation and divorce or are simply less likely to re-marry. Nonetheless, across both datasets, older adults who were currently married consistently demonstrated the lowest prevalence of cognitive disability compared to other marital status groups. These findings align with the study by Lee [28], which reported that being married was associated with lower odds of cognitive impairment. Marriage, according to the authors, provides critical social support, companionship, and opportunities for engagement in mentally stimulating activities, all of which contribute to better cognitive health. Identifying marital status as a risk factor highlights the importance of developing targeted interventions and support systems to promote healthy cognitive ageing among older adults.

Another notable finding in this study was the relationship between employment status and self-reported cognitive disability. In both 2017 and 2022, not being employed was significantly associated with higher odds of reporting cognitive disability. Several factors may explain this association. Older adults often face difficulties securing or maintaining employment due to outdated legal definitions of retirement age, labelling and ageist discrimination (such as assumptions that older adults no longer wish to work or are incapable of doing so), as well as negative attitudes linked to disability, race, ethnicity, and gender. Additionally, there is a pervasive belief that older individuals possess sufficient financial resources to sustain a comfortable lifestyle, thereby negating the perceived need for employment [29]. Another contributing factor could be that persons with disabilities are frequently excluded from consideration as potential members of the workforce [30], leading to higher unemployment rates among this group. Societal perceptions, fear, myths, and prejudice continue to hinder the full inclusion of persons with disabilities in economic activities globally [31]. Common myths persist, such as the misconception that persons with disabilities are incapable of working or that accommodating them is prohibitively costly [32]. Supporting these findings, Emerson [33] concluded that individuals classified as economically inactive were significantly more likely to be disabled across all age groups. These observations underscore the complex interplay between disability, employment status, and social perceptions, highlighting the need for inclusive employment policies and practices that promote the participation of older adults with disabilities in the workforce.

The environments in which individuals live profoundly influence their health outcomes, disease experiences, and overall well-being [34]. The findings of this study revealed that older adults residing in rural areas had the highest odds of reporting cognitive disability, followed by those living in urban villages and, lastly, those in cities and towns, in both 2017 and 2022. Several factors may explain this pattern. One possibility is that older adults often migrate to rural areas upon retirement or after the onset of a disability or severe chronic health condition, thereby concentrating a higher proportion of individuals with disabilities in these regions [35]. Additionally, rural–urban disparities in socio-economic status and healthcare access likely contribute to these findings, as older adults in rural areas generally face greater disadvantages [36]. Rural communities are often more geographically isolated and typically have fewer transportation options and more limited access to healthcare facilities compared to urban settings. Consequently, the coordination and delivery of care for individuals with disabilities may be significantly more challenging for rural residents due to these systemic geographic and logistical barriers. These results underscore the critical need for targeted interventions to address healthcare inequities and to improve support services for older adults with disabilities living in rural areas.

The analysis further revealed that, across all three types of residence, (cities and towns, urban villages, and rural areas), the prevalence of disabilities among older adults was consistently higher among females than males in both 2017 and 2022. This pattern may be attributed to the heightened vulnerabilities and reduced protection experienced by women, particularly in rural contexts [37]. Women with disabilities living in rural areas often face compounded disadvantages, including limited access to education, employment opportunities, income, social rights, welfare benefits, and public transportation. These challenges are further exacerbated by environmental and architectural barriers that restrict mobility and independence [38]. Such structural inequalities likely contribute to the disproportionately higher rates of disability observed among older women across all residential settings.

Importantly, few studies in Botswana have systematically examined the predictive role of existing physical disabilities in cognitive decline among ageing populations. The findings of this study demonstrates that older adults who already had a physical disability (such as difficulties in walking, seeing, hearing, or self-care) were at markedly higher risk of reporting cognitive disability over time compared to their counterparts without such conditions. The association suggests a health burden, where existing physical impairments may exacerbate vulnerability on cognitive deterioration. Although formal statistical tests for trend were not conducted, descriptive comparisons showed a marked increase in cognitive disability prevalence over the five-year period, underscoring the need for integrated health strategies as Botswana’s population continues to age.

## Limitations of the study

This study had several limitations that must be acknowledged. First, it did not incorporate important health-related variables such as smoking status, physical activity levels, body mass index (BMI), alcohol consumption, and the number of chronic diseases. The analysis was confined to the variables available in the 2022 Botswana Population and Housing Census, which restricted the inclusion of these critical factors due to the absence of relevant data. Future research would benefit from a more comprehensive and systematic examination of how chronic diseases, health behaviours, and lifestyle factors influence functional disability, mobility impairment, and cognitive disability among older adults.

Second, the study primarily relied on cross-sectional census data, which, although extensive and nationally representative, lacks the depth provided by longitudinal studies that track changes in individual health status over time. Consequently, while the study identified significant associations between sociodemographic factors and cognitive disability, it cannot establish causal relationships. As with all cross-sectional designs, interpretations of temporal causality are limited [39].

To build upon these findings, future studies should prioritize longitudinal research designs and incorporate qualitative approaches to capture the lived experiences of older adults with cognitive disabilities. Special attention should be given to rural and socio-economically disadvantaged populations, where vulnerabilities may be more pronounced. Additionally, investigating the role of healthcare access and quality in shaping cognitive health outcomes would offer valuable insights for informing targeted interventions aimed at reducing cognitive disability rates in Botswana’s ageing population.

Another key limitation of this study lies in the potential inconsistencies in field implementation between the 2017 BDS and 2022 PHC, which may have influenced the comparability of results across time. Variations in interviewer training, question phrasing, and respondent understanding could have contributed to differences in how cognitive disability was reported. While the statistical analyses conducted are robust and the findings offer important insights, they should be interpreted with caution due to these potential methodological and reporting limitations. This caveat is important to avoid overgeneralization of the results and underscores the need for further targeted and methodologically harmonized research to validate and expand upon the study’s findings.

## Conclusion

This study documents a substantial rise in the prevalence of self-reported cognitive disability among older adults in Botswana between 2017 and 2022, with consistent increases observed across all sociodemographic groups. While measurement differences, such as the use of proxy reporting and variations in survey administration, may have influenced these findings, broader societal changes, including increased public awareness and reduced stigma surrounding disability, likely encouraged greater self-identification. Furthermore, demographic shifts towards an ageing population, the growing burden of non-communicable diseases, and the potential neurological impacts of the COVID-19 pandemic suggest that the observed increase may, at least in part, reflect a genuine rise in cognitive impairment. An additional finding of interest was the anomalous pattern in the 2017 data, where cognitive disability prevalence did not consistently rise with age, contrary to established trends in the literature. This irregularity may be attributable to data quality issues, including underreporting or misclassification among the older adults.

Addressing the growing challenge of cognitive disability among older adults in Botswana requires the implementation of targeted, context-sensitive intervention programs, particularly for rural and socio-economically disadvantaged populations. These initiatives should prioritize education on cognitive health, alongside the expansion of resources to improve access to essential healthcare services. Integrating routine cognitive health screenings into primary healthcare settings is strongly recommended, with special attention given to older adults experiencing physical impairments such as hearing, mobility, vision, or self-care difficulties. Furthermore, healthcare and social service delivery must be adapted to meet the specific needs of individuals in rural areas and those with lower educational attainment, emphasizing health literacy and ensuring the accessibility and affordability of medical services.

Given the projected rise in disability prevalence, the development of a sustainable long-term care (LTC) system is critical. Such a system should be designed to support both family caregivers and formal healthcare structures, equipping them to meet the anticipated increase in demand. In addition, community-based awareness campaigns that promote cognitive disability prevention through physical activity, social participation, and healthy lifestyle practices are essential, particularly for high-risk groups [40]. Together, these strategies could offer a comprehensive approach to enhancing cognitive health outcomes and strengthening support systems for ageing populations in Botswana.

## Supplementary Information

Below is the link to the electronic supplementary material.

Supplementary Material 1

## Data Availability

The data supporting the findings of this study are accessible through Statistics Botswana and can be obtained by researchers upon request. Additionally, the data may be provided by the authors upon reasonable request, subject to approval from Statistics Botswana.
