# Supplementary material for: Cognitive disability among older adults in Botswana: prevalence, trends, and sociodemographic determinants from cross-sectional data
Source: BMC Geriatr. 2025 Aug 29;25:670. doi: 10.1186/s12877-025-06383-w (PMC12395718; doi:10.1186/s12877-025-06383-w)
Supplement: Supplementary file 1 — Supplementary Material 1 [file 12877_2025_6383_MOESM1_ESM.doc]

STROBE Statement: Checklist of items that should be included in reports of ***cross-sectional studies***

**Cognitive Disability among Older Adults in Botswana: Prevalence, Trends, and Sociodemographic Determinants from Cross-Sectional Data**

| **Item** | **Recommendation** | **How the Study Meets It** |
| --- | --- | --- |
| **Title and Abstract** |  |  |
| 1(a) | Indicate the study’s design | - The title and abstract clearly describes the design as a cross-sectional study using national survey data. |
| 1(b) | Balanced summary in abstract | - The abstract succinctly outlines background, methods, key results, and implications. |
| **Introduction** |  |  |
| 2 | Background and rationale | - Well-articulated. Introduces ageing and disability in Botswana and the need for analysis using 2017 BDS and 2022 Census data. |
| 3 | Objectives and hypotheses | - Objectives are clearly stated: to examine prevalence, trends, and sociodemographic determinants of cognitive disability. |
| **Methods** |  |  |
| 4 | Study design | - Secondary analysis of the Botswana Demographic Survey (BDS) 2017 and 2022 Census. |
| 5 | Setting, location, dates | - National setting, 2017 and 2022 surveys. Mentioned in data section. |
| 6(a) | Participants: criteria and selection | - Eligibility included individuals aged 65+; selection followed a two-stage probability design for the 2017 BDS. For census 2022 is was a complete enumeration. |
| 7 | Variables and definitions | - Cognitive disability was defined using standardized questions; independent variables included age, gender, residence, etc. |
| 8 | Data sources/measurement | - Data sourced from 2017 BDS and 2022 Census. Measurement criteria for cognitive disability and other variables described. |
| 9 | Bias | - Discussion of underreporting bias and limitations of self-reported data is noted in the discussion. |
| 10 | Study size | - Weighted sample of 118,060 for the 2017 BDS; explanation clarified in methods. Similarly, for the 2022 Botswana Population and Housing Census (PHC), 130,551 older adults were identified from the complete enumeration |
| 11 | Quantitative variables | - Clearly grouped (e.g., age in bands), and categories. |
| 12(a) | Statistical methods | - Multivariate logistic regression described; controlled for confounders. |
| 12(b) | Subgroup analyses | - Trends were stratified by background characteristics. |
| 12(c) | Missing data | - Participants with missing cognitive disability data were excluded; explained in methods. |
| 12(d) | Sampling strategy | - Weighted data are used and design weights are applied in regression. |
| 12(e) | Sensitivity analyses | - N/A |
| **Results** |  |  |
| 13(a) | Participant numbers at each stage | - Weighted estimates given; number of older adult respondents clarified in methods and results. |
| 13(b) | Reasons for non-participation | - N/A |
| 13(c) | Flow diagram | - N/A |
| 14(a) | Descriptive data | - Demographic characteristics (Table 2) presented by survey year. |
| 14(b) | Missing data | - Minimal missing data mentioned; cases with missing cognitive disability were excluded. (Table 1) |
| 15 | Outcome data | - Prevalence figures and logistic regression outputs provided. |
| 16(a) | Estimates with precision/confounders | - Adjusted odds ratios with 95% CIs; confounders listed. |
| 16(b) | Category boundaries | - Reported for all grouped variables. |
| 16(c) | Translation into absolute risk | - N/A |
| 17 | Other analyses | - Trend analysis across time periods (2017 vs. 2022). |
| **Discussion** |  |  |
| 18 | Key results | - Clearly summarized with links to objectives. |
| 19 | Limitations | - Includes sampling, self-report bias, and methodological limitations. |
| 20 | Interpretation | - Balanced and cautious interpretation in light of limitations. |
| 21 | Generalisability | - Addressed in discussion: findings generalized to national older population in Botswana. |
| **Other Information** |  |  |
| 22 | Funding | - Funding details provided: No Funding. |
